# Effects of Oil and Processing Conditions on Formation of Heterocyclic Amines and Polycyclic Aromatic Hydrocarbons in Pork Fiber

**DOI:** 10.3390/foods12183504

**Published:** 2023-09-20

**Authors:** Yu-Wen Lai, Baskaran Stephen Inbaraj, Bing-Huei Chen

**Affiliations:** 1 Department of Food Science, Fu Jen Catholic University, New Taipei City 242062, Taiwan; linda871123@gmail.com (Y.-W.L.); sinbaraj@yahoo.com (B.S.I.); 2 Department of Nutrition, China Medical University, Taichung 40402, Taiwan

**Keywords:** heterocyclic amines (HAs), polycyclic aromatic hydrocarbons (PAHs), fried pork fiber, QuEChERS, UPLC-MS/MS, GC-MS/MS

## Abstract

Toxic compounds such as heterocyclic amines (HAs) and polycyclic aromatic hydrocarbons (PAHs) can be produced during food processing, especially meat products. This study aims to monitor the formation of HAs and PAHs in fried pork fiber, a common meat product in Taiwan, at different processing conditions. A total of six experimental groups, including raw pork tenderloin, dried pork filaments, sesame oil-stir-fried pork at 160 °C for 15 min, sesame oil-stir-fried pork at 200 °C for 6 min, lard-stir-fried pork at 160 °C for 15 min, and lard-stir-fried pork at 200 °C for 6 min, were prepared and analyzed for formation of HAs via UPLC-MS/MS and PAHs via GC-MS/MS in triplicate. Frying in sesame oil or lard showed a greater content of total HAs in fried pork fiber processed at 160 °C for 15 min than at 200 °C for 6 min. However, in the same heating conditions, pork fiber fried in sesame oil produced a higher level of total HAs than that fried in lard. Of the various HAs in fried pork fiber, both Harman and Norharman were generated in the highest amount. The precursors, including reducing sugar, amino acid, and creatine/creatinine, played a vital role in HAs formation in fried pork fiber. For total PAHs, the highest level was shown for pork fiber fried in lard at 200 °C/6 min, followed by frying in sesame oil at 200 °C/6 min and 160 °C/15 min, and in lard at 160 °C/15 min. Like HAs, at the same heating condition, a greater content of total PAHs was produced in pork fiber fried in sesame oil than in lard. Notably, the highly toxic benzo[a]pyrene was undetected in fried pork fiber. The PAH precursor benzaldehyde was shown to generate at a much higher level than 2-cyclohexene-1-one and trans,trans-2,4-decadienal in fried pork fiber, and it should play a more important role in PAH formation. Principal component analysis (PCA) also revealed that the formation mechanism of HAs and PAHs in fried pork fiber was different.

## 1. Introduction

Toxins such as heterocyclic amines (HAs) and polycyclic aromatic hydrocarbons (PAHs) can be produced at varying types and levels during the processing of meat products. HAs are further divided into thermic HAs and pyrolytic HAs, with the former being composed of imidazoquinolines (IQ, iso-IQ and MeIQ), imidazoquinoxalines (Iqx, 8-MeIQx, 7,8-DiMeIQx, 4,8-DiMeIQx), and imidazopyridines (PhIP, DMIP, IFP), while the latter consists of α-carbolines (AαC, MeAαC), β-carbolines (Harman, Norharman), γ-carbolines (Trp-P-1, Trp-P-2), δ-carbolines (Glu-P-1, Glu-P-2), phenylpyridines (Phe-P-1), tetraazafluoranthenes (4-amino-6-methyl-1H-2,5,10,10b-tetraazafluoranthene-3-amine, Orn-P-1), benzimidazoles (4-amino-1,6-dimethyl-2-methylamino-1H,6H-pyrrolo-[3,4-f]benzimidazole-5,7-dione, Cre-P-1), and carbazoles (3,4-cyclopenteno-pyrido[3,2-a]carbazole, Lys-P-1) [1]. Thermic HAs are formed from amino acid, creatine/creatinine, and hexose via a Maillard browning reaction at 100–300 °C, while pyrolytic HAs are produced via the degradation of protein/amino acid at >300 °C [2]. Based on the categorization by the International Agency of Research on Cancer [3], IQ is a probable human carcinogen (group 2A), and MeIQ, 8-MeIQx, AαC, Try-P-1, Try-P-2, Glu-P-1, Glu-P-2, PhIP, and MeAαC are possible human carcinogens (group 2B). The full names of all the HA compounds are shown in Table S1.

Likewise, PAHs are divided into high-molecular-weight PAHs with ≥3 benzene rings and low-molecular-weight PAHs with <3 benzene rings, with the former being more stable and possessing greater toxicity than the latter [4]. PAHs are usually generated through the carbonization or incomplete combustion/pyrolysis of organic materials and accumulate in the food chain owing to their structural stability [5]. At temperatures >200 °C, the combustion of fat, protein, and carbohydrate can lead to PAH formation through dehydrogenation and bond breakage, while they are produced through polymerization at lower temperatures [6,7]. On the other hand, PAHs can be produced via carbonization at <200 °C and high pressure through substance decay [5]. Based on a report by IARC [8], BaP was classified as carcinogenic to humans (group 1), DBahA, CcdP, and DBalP as probable human carcinogens (group 2A), and NaP, BaA, CHR, BbF, IP, BjF, MCH, DBaiP, DBahP, and BkFL (benzo[k]fluoranthene) as possible human carcinogens (group 2B), while the other PAHs, including AcP, Flu, Phe, Ant, FL, Pyr, BghiP, BcF, and DBaeP, were classified as not carcinogenic to humans (group 3). The full names of all the PAH compounds are shown in Table S2.

The formation and inhibition of HAs and PAHs during meat processing have recently been the focus of intense research. For instance, Lai et al. [9] studied the formation and inhibition of HAs and PAHs in pork jerky and reported that a greater content of PAHs was generated at 220 °C than at 180 °C during roasting, but the HA formation remained unaffected, which can be attributed to the difference in formation mechanism, as demonstrated via principal component analysis. Moreover, the addition of sugar could reduce the generation of both HAs and PAHs during roasting, while soy sauce minimized PAH formation and promoted HA formation. In another study dealing with the formation and inhibition of HAs and PAHs in ground pork during marination, a time-dependent rise in both HA and PAH contents was shown, with HAs being more prone to formation than PAHs [10]. Additionally, the incorporation of cinnamon powder (0.5%) or green tea powder (0.5%) was effective in preventing the formation of HAs and PAHs in pork during marination.

Owing to their presence in trace amounts (ppb), the analysis of HAs and PAHs in meat products has been strenuous. QuEChERS is an effective extraction method successfully applied for various analytes from different food matrices, which involves the addition of inorganic salt for separating the organic solvent from water, followed by purification from interfering matrices using adsorbents, and it has been successfully used for the simultaneous extraction and purification of HAs and PAHs in processed meat products with high accuracy and precision [9,10]. Therefore, in this study, we also employed the QuEChERS as the sample preparation method for both HAs and PAHs from raw pork, dried pork filaments, and fried pork fiber, followed by identification and quantitation using UPLC-MS/MS for HAs and GC-MS/MS for PAHs.

Fried pork fiber, a common meat byproduct sold in Taiwan, can be produced by marinating raw pork tenderloin, smashing it to filaments, heating it to dryness, stir-frying it, and then adding edible oil for continuous stir-frying until a delicious-tasting crispy product is obtained. As the processing of fried pork fiber involves high temperatures and long heating times, a high content of HAs and PAHs can be generated. Thus, it is imperative to minimize the formation of HAs and PAHs in fried pork fiber by controlling the processing conditions. This study aims to monitor the formation of HAs and PAHs in fried pork fiber as affected by frying temperature, time length, and oil type.

## 2. Materials and Methods

### 2.1. Processing of Fried Pork Fiber

**Figure 1:**
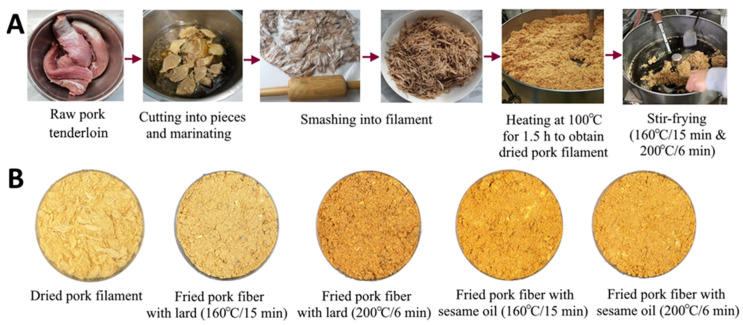
Processing steps of fried pork fiber (**A**) and appearance of products (**B**).

One kg of raw pork tenderloin (raw pork) was collected, cut into pieces, and mixed with 2 kg of marinated juice containing 200 g of soy sauce (10%), 20 g of crystal sugar (1%), and 1780 g of distilled water (89%). After cooking for 4 h at 96 °C, this mixture was cooled and smashed with a wooden mallet to produce thread-like pork filament and poured into a frying pan for heating at 100 °C for 1.5 h to obtain dried pork filaments. Then, 200 g of dried pork filaments were poured into a fryer separately for stir-frying at 160 °C for 15 min or 200 °C for 6 min, with 28 g of sesame oil (14%) or pork lard (14%) being added 5 min before the end of the low-temperature long-time stir-frying process (160 °C/15 min) and 3 min before the end of the high-temperature short-time stir-frying process (200 °C/6 min). The most optimal addition time for sesame oil or pork lard has to be controlled for different frying conditions to maintain the crispiness of fried pork fiber. Figure 1 shows the different processing steps in the preparation of fried pork fiber (A) and the appearance of products obtained (B). The sensory evaluation was conducted by 10 graduate students in our department, and this fried pork fiber product was deemed to be acceptable in terms of color, flavor, and texture. Next, the raw pork, dried pork filament, and fried pork fiber were subjected to HA and PAH analysis via UPLC-MS/MS and GC-MS/MS, respectively. The marinated juice composition and frying condition were selected as they were most often used for processing fried pork fiber in Taiwan.

### 2.2. Simultaneous Extraction and Purification of HAs and PAHs in Raw Pork, Dried Pork Filament and Fried Pork Fiber

Simultaneous extraction and purification of HAs and PAHs in raw pork, dried pork filament, and fried pork fiber were based on a method reported by Lai et al. [9,10]. Initially, a 2-g pork sample was taken in a centrifuge tube (50 mL) and mixed with a ceramic homogenizer, after which deionized water (10 mL) was added and the mixture was shaken for 10 min, followed by adding 10 mL 1% acetic acid in acetonitrile, shaking for 10 min, adding the extraction powder (4 g of magnesium sulfate and 1 g of sodium acetate), vortexing for 1 min, centrifuging for 10 min (4000 rpm) at 4 °C, and collecting the supernatant (6 mL) for purification. Next, the supernatant was added to a 15-mL centrifuge tube containing 300 mg of PSA, 900 mg of magnesium sulfate, and 300 mg of C18 EC, followed by vortexing for 1 min, centrifuging for 10 min at 4 °C (4000 rpm), collecting 1 mL of the supernatant, evaporating under nitrogen, dissolving in 0.2 mL of hexane containing internal standard (Triphenylene) at 10 ppb, and filtering with a 0.22-μm membrane filter for PAH analysis via GC-MS/MS. For HA analysis, the residue was dissolved in 0.2 mL of methanol containing internal standard (4,7,8-TriMeIQx) at 1 ppb for quantitation via UPLC-MS/MS analysis.

### 2.3. Separation, Identification and Quantitation of HAs in Raw Pork, Dried Pork Filament and Fried Pork Fiber by UPLC-MS/MS

A method reported by Lai et al. [9,10] was used for separation, identification, and quantitation of HAs in raw pork, dried pork filament, and fried pork fiber via UPLC-MS/MS. An ACQUITY BEH C18 column with dimensions of 50 × 2.1 mm ID, 1.7 μm particle size, and a gradient mobile phase consisting of 20 mM ammonium acetate (pH 4.5) (A) and acetonitrile (B) was used for separation of 21 HAs with 95% A and 5% B in the beginning, held for 2 min, raised to 90% B in 3.3 min, held for 4 min, and returned to the initial ratio in 4.1 min, with the flow rate at 0.7 mL/min and column temperature at 30 °C. The HAs detection in raw pork, dried pork filament, and fried pork fiber was conducted in the selective reactive monitoring (SRM) mode by using the same precursor ions and product ions reported for identification and quantitation by Lai et al. [9,10], while the details on the method validation of HAs and the matrix effect are reported in our previous study [9].

### 2.4. Separation, Identification and Quantitation of PAHs in Raw Pork, Dried Pork Filament and Fried Pork Fiber by GC-MS/MS

The method reported by Lai et al. [9,10] was adopted to determine PAHs in raw pork, dried pork filament, and fried pork fiber via GC-MS/MS. By using an Agilent DB-5MS capillary column with dimensions of 30 m × 0.25 mm ID and 0.25 μm film thickness, 23 PAHs could be separated within 78 min, with helium as a carrier gas, at a flow rate at 1.25 mL/min, injection temperature at 320 °C (splitless mode), and MS interface temperature (280 °C). The following temperature programming was used: 80 °C initially, maintained for one min, increased to 200 °C at 5 °C/min, maintained for 10 min, increased to 220 °C at 5 °C/min, maintained for 5 min, increased to 230 °C at 1 °C /min, maintained for 10 min, increased to 320 °C at 10 °C/min, and maintained for 10 min. Similar to HAs, the SRM mode was used for detection of PAHs in raw pork, dried pork filament, and fried pork fiber. For MS detection, the same precursor and product ions as those reported by Lai et al. [9,10] were used for identification and quantitation, while the details on the method validation of PAHs and the matrix effect are reported in a previous study by Lai et al. [9].

### 2.5. Determination of HA Precursors

#### 2.5.1. Reducing Sugar

A method reported by Chen et al. [11] was used to determine the reducing sugar contents in raw pork, dried pork filament, and fried pork fiber. Initially dinitrosalicyclic acid (0.315 g) was dissolved in distilled water (50 mL), and 10 mL of sodium hydroxide solution (0.2 g/mL) was added, after which sodium potassium tartrate (9.1 g) was added to this mixture, followed by diluting to 100 mL with distilled water, collecting one mL, mixing with 0.1 mL of five glucose standard concentrations (1.25, 2.5, 5, 8, 10 mg/mL) separately for preparation of the glucose standard curve by heating at 100 °C for 10 min, and measuring absorbance at 570 nm after cooling. Similarly, the glucose contents in raw pork, dried pork filament, and fried pork fiber were calculated based on the linear regression equation obtained from the glucose standard curve. Next, a 2-g sample was collected and mixed with 10-mL of distilled water, followed by shaking (60 min), centrifuging (4000 rpm, 20 min), collecting the supernatant, diluting to 50 mL with distilled water, collecting 0.1 mL and mixing with 1 mL of dinitrosalicylic acid, heating at 100 °C for 10 min, and measuring absorbance at 570 nm after cooling.

#### 2.5.2. Amino Acid

The amino acid contents in raw pork, dried pork filament, and fried pork fiber were measured based on a report by TFDA [12]. Initially, the derivatized amino acid standard solution was prepared by dissolving each amino acid (37.5 mg) in 1 N hydrochloric acid (10 mL), and 20 μL was collected for mixing with 0.4 M of boric acid buffer solution (100 μL), after which this solution was mixed thoroughly and phthaldehyde (20 μL) was added for vortexing for 60 s, followed by adding 9-fluorenylmethyl chloroformate (20 μL), vortexing for 30 s and adding deionized water (1280 μL) for mixing thoroughly. Next, a 0.5-g sample of raw pork, dried pork filament, or fried pork fiber was mixed with 20 mL of 0.1 N hydrochloric acid, followed by shaking with sonication for 10 min, dilution with 0.1 N HCl (25 mL), filtration through a membrane filter, collecting a portion (20 μL) for mixing with 0.4 M of boric acid buffer solution (100 μL), adding 9-fluorenylmethyl chloroformate (20 μL), vortexing for 30 s, and adding deionized water (1280 μL) for HPLC analysis using a Poroshell HPH-C18 column (10 cm × 3.0 mm ID, particle size 2.7 μm) with a column temperature of 40 °C, an injection volume of 10 μL, a flow rate of 0.5 mL/min, and a detection wavelength at 262 nm and 338 nm. A gradient mobile phase of 40 mM of sodium dihydrogen phosphate (pH 7.8) (A) and acetonitrile/methanol/water (45:45:10, *v*/*v*/*v*) (B) was used to separate various amino acids in pork samples, while identification was performed by comparing the retention times and absorption spectra of unknown peaks with reference standards for subsequent quantitation using the formula described in a report by TFDA [12].

#### 2.5.3. Creatine and Creatinine

Both creatine and creatinine contents in raw pork, dried pork filament, and fried pork fiber were measured by adopting a method reported by Gibis and Loeffler [13]. In brief, a 20-g pork sample was collected and mixed with distilled water (100 mL), followed by homogenizing this mixture at 24,000 rpm for 2 min, standing at 18 °C for 20 min, filtration through a filter paper, adding perchloric acid (1 moL/L), neutralizing pH to 6.5 with potassium hydroxide, and using the assay kits to determine creatine and creatinine contents in pork samples.

### 2.6. Determination of PAH Precursors

To determine PAH precursors, including 4,4-dimethyl-2-cyclohexene-1-one, cyclohexene, 2-cyclohexene-1-one, benzaldehyde, and trans,trans,2,4-decadienal in raw pork, dried pork filament, and fried pork fiber, two methods, reported by Bueno et al. [14] and Lai et al. [9], were adopted by initially pouring a 0.5-g of pork samples into a headspace vial (20 mL) and mixing with water (2.5 mL), after which this solution was heated at 65 °C for 10 min. Then, the fiber head was inserted into the sample vial for exposure to the headspace at 65 °C for 20 min for solid phase microextraction, followed by insertion into the GC-MS inlet (Agilent 7890B and 5977A, Santa Clara, CA, USA) for analysis. Next, the fiber was desorbed in the injection port at 260 °C for one min with a splitless mode.

For separation of PAH precursors, an Agilent HP-5MS column (Agilent Technologies, CA, USA) with dimensions of 30 m × 0.25 mm ID and film thickness of 0.25 μm was used. The column temperature programming was 40 °C initially, maintained for 4 min, increased to 50 °C at 5 °C/min, maintained for 2 min, raised further to 120 °C at 5 °C/min, maintained for 3 min, and then increased to 260 °C at 30 °C/min, and maintained for 5 min. Helium was used as the carrier gas with a flow rate of 1 mL/min. MS detection was conducted in electron ionization mode with ionization voltage at 70 eV, and, by using the selective ion monitoring (SIM) mode, 2-cyclohexene-1-one, benzaldehyde, and trans,trans-2,4-decadienal were identified at m/z 68, 105, and 81, respectively. Then, their levels (ng/g) in samples were determined by using the linear regression equation obtained from the respective standard curves prepared with their standard concentration ranging from 0.1 to 20 ng/mL, 4 to 100 ng/mL, and 0.1 to 800 ng/mL. However, both 4,4-dimethyl-2-cyclohexene-1-one and cyclohexene were not quantified as they were not detected in raw pork, dried pork filament, and fried pork fiber by GC-MS.

### 2.7. Principal Component Analysis (PCA)

The mean data obtained via triplicate analyses was subjected to PCA through clustering HAs and PAHs content data obtained under various processing methods. Initially, a group of correlated variables was converted into a new group of linearly noncorrelated variables according to an eigen value >1. Then, the PCA was run on Origin^®^ 2019b vision 9.65 (Northampton, MA, USA) software with a Kaiser–Meyer–Olkin value of 0.80 and *p* < 0.05 to establish the possible relationship between the formation of HAs and PAHs in fried pork fiber during processing under different processing conditions, including oil type (sesame oil and lard) and stir-frying temperature (160 °C/15 min and 200 °C/6 min).

### 2.8. Statistical Analysis

A total of 6 experimental groups, including raw pork, dried pork filaments, sesame oil-stir-fried pork at 160 °C for 15 min, sesame oil-stir-fried pork at 200 °C for 6 min, lard-stir-fried pork at 160 °C for 15 min, and lard-stir-fried pork at 200 °C for 6 min, were prepared and analyzed for formation of HAs via UPLC-MS/MS and PAHs via GC-MS/MS. Each set of experiments was replicated in triplicate and the mean values were compared for significance at α = 0.05 via analysis of variance (ANOVA) and Duncan’s multiple range test using the Statistical Analysis System 9.4 software [15]. In addition, a two-factorial design analysis was conducted using the two-way ANOVA method to elucidate the independent contributions of oil type (sesame oil and lard), frying conditions (160 °C/15 min and 200 °C/6 min), and their interaction (oil type × frying condition) on HAs and PAHs formation.

## 3. Results and Discussion

### 3.1. Separation of HA Standards via UPLC-MS/MS and PAH Standards via GC-MS/MS

**Figure 2:**
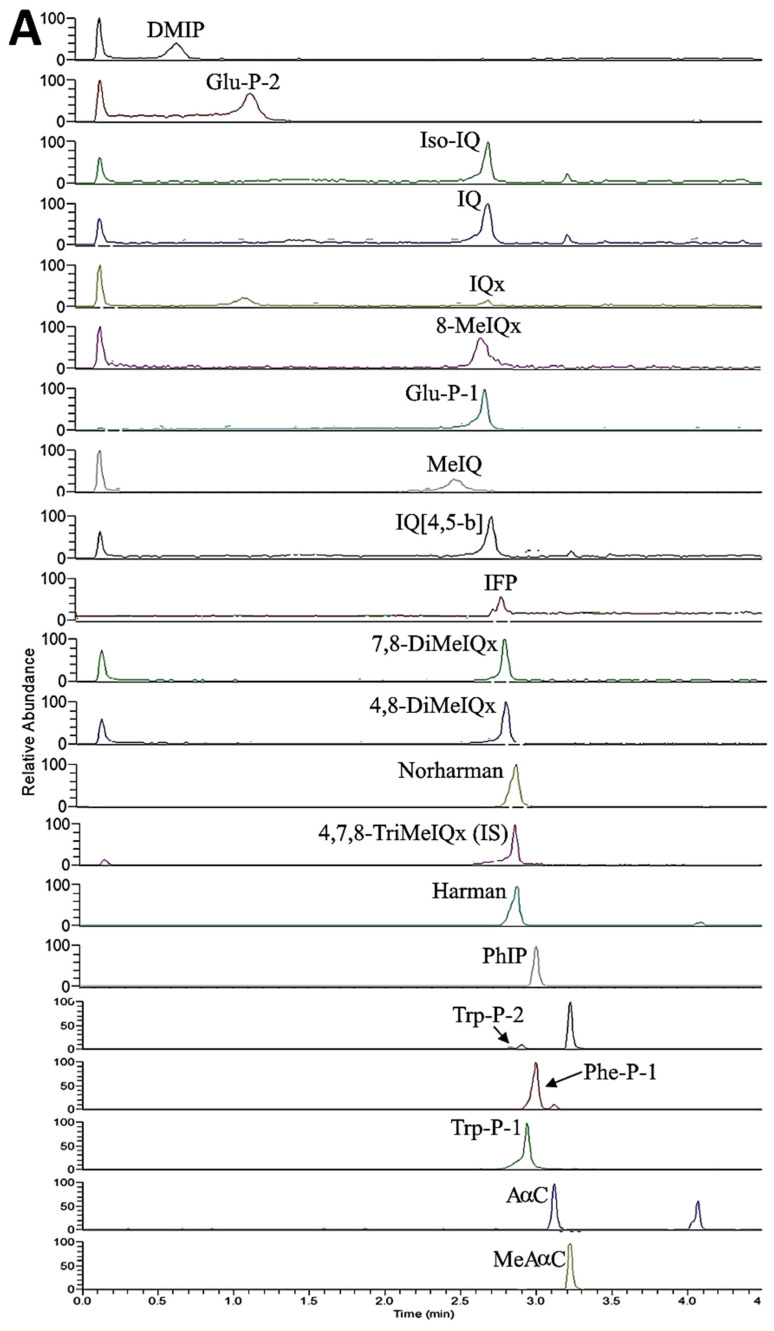

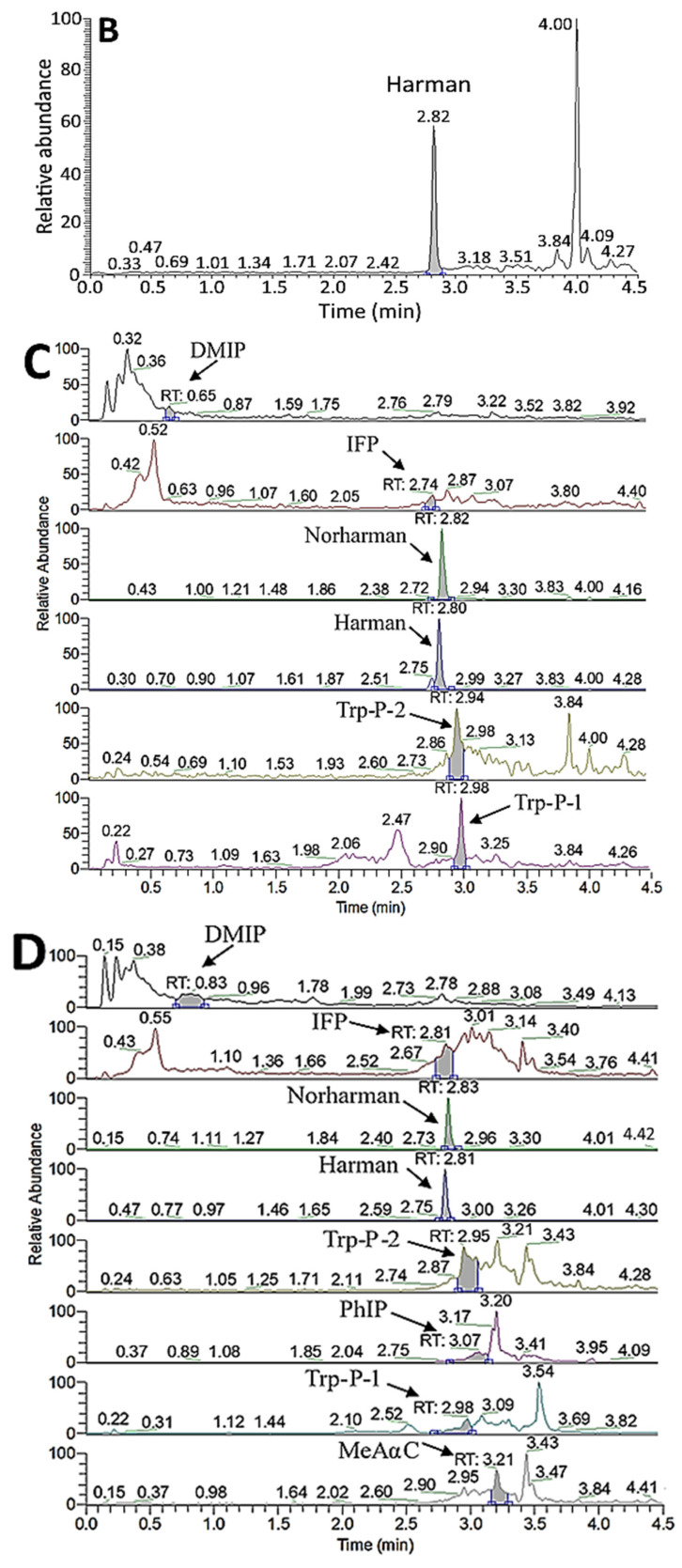

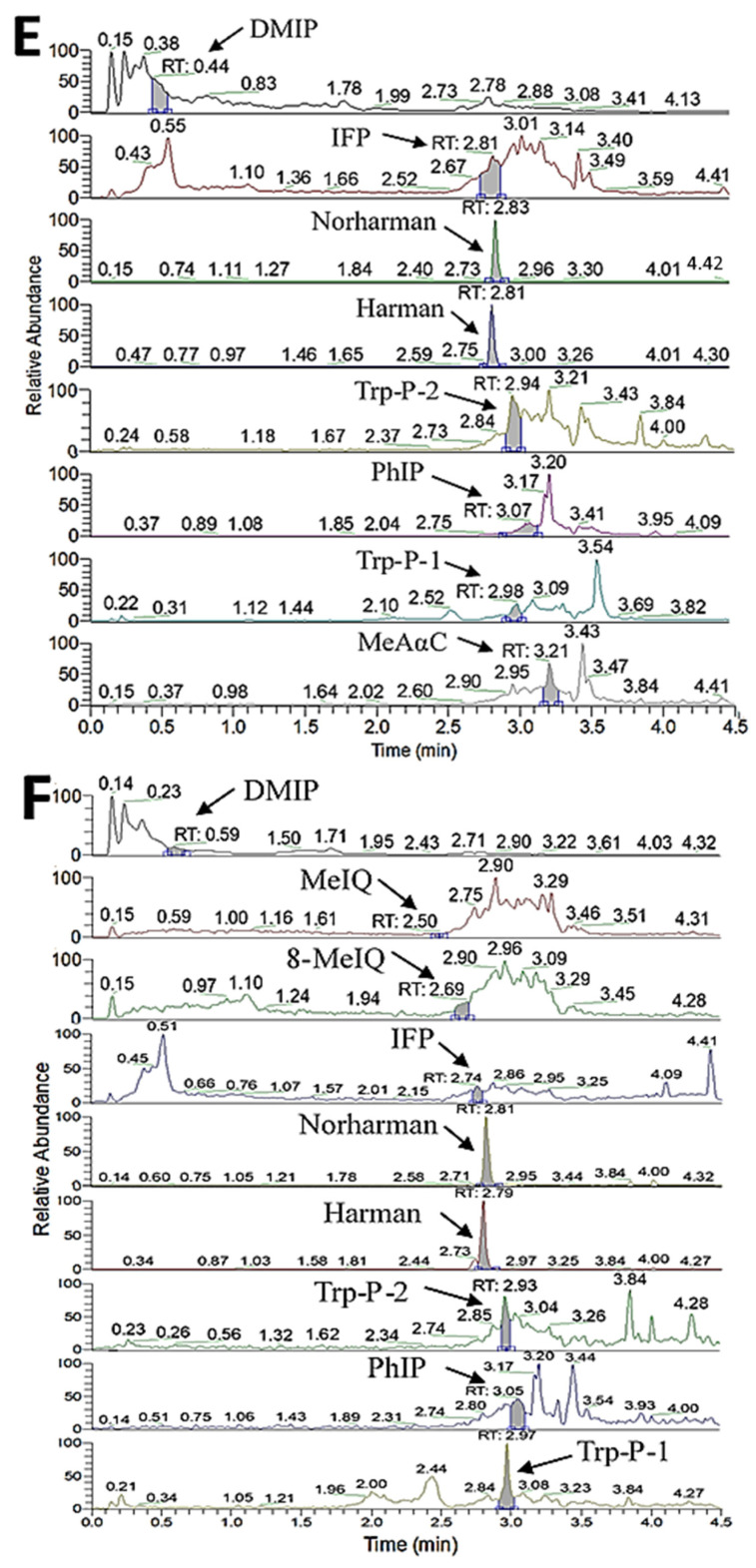

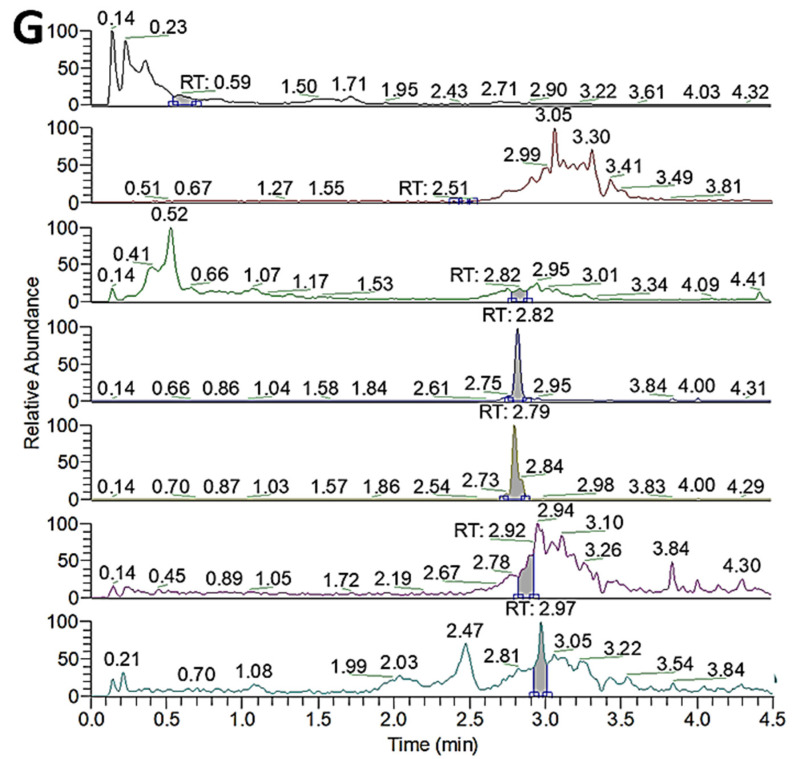
Chromatograms of HA standards (**A**), as well as HAs in raw pork (**B**), dried pork filaments (**C**), fried pork fiber (sesame oil, 160 °C/15 min) (**D**), fried pork fiber (sesame oil, 200 °C/6 min) (**E**), fried pork fiber (lard, 160 °C/15 min) (**F**), and fried pork fiber (lard, 200 °C/6 min) (**G**) in SRM mode via UPLC-MS/MS. The full names of the abbreviated HAs are provided in Table S1.

**Figure 3:**
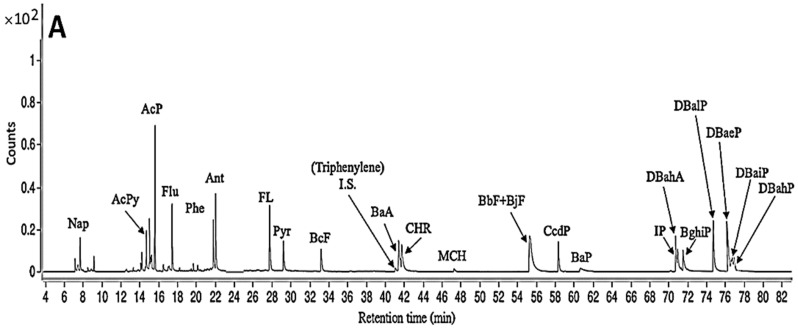

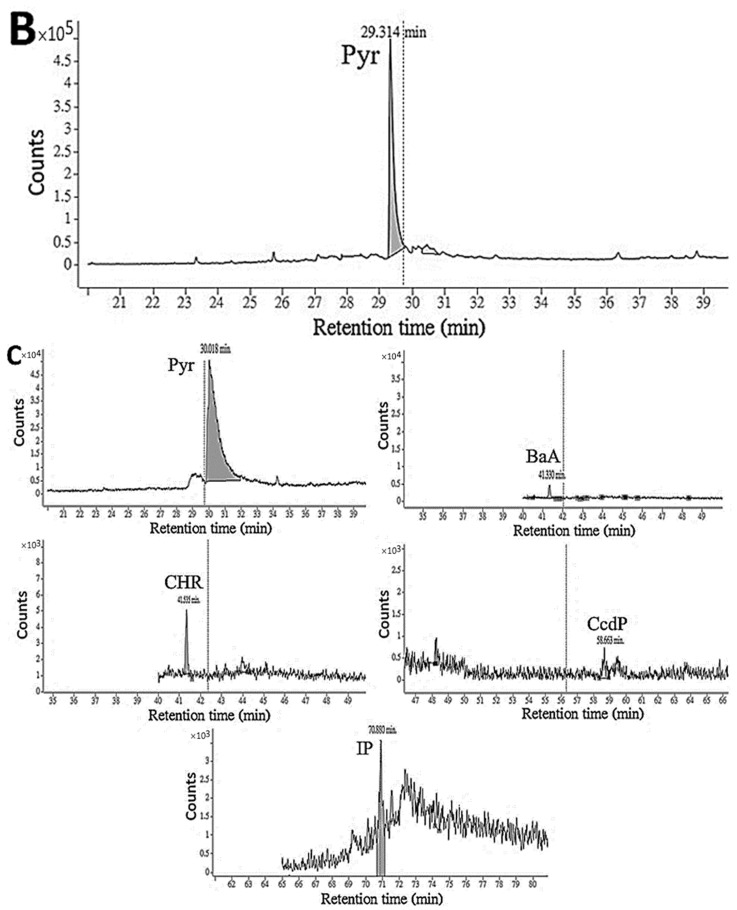

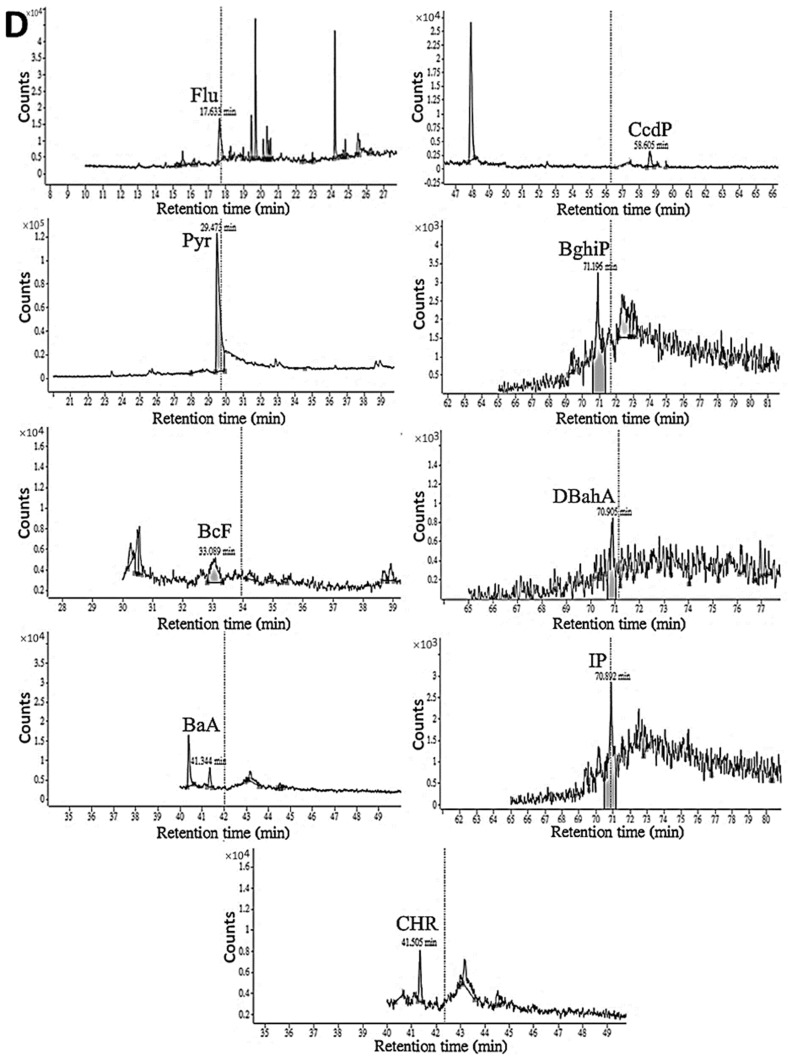

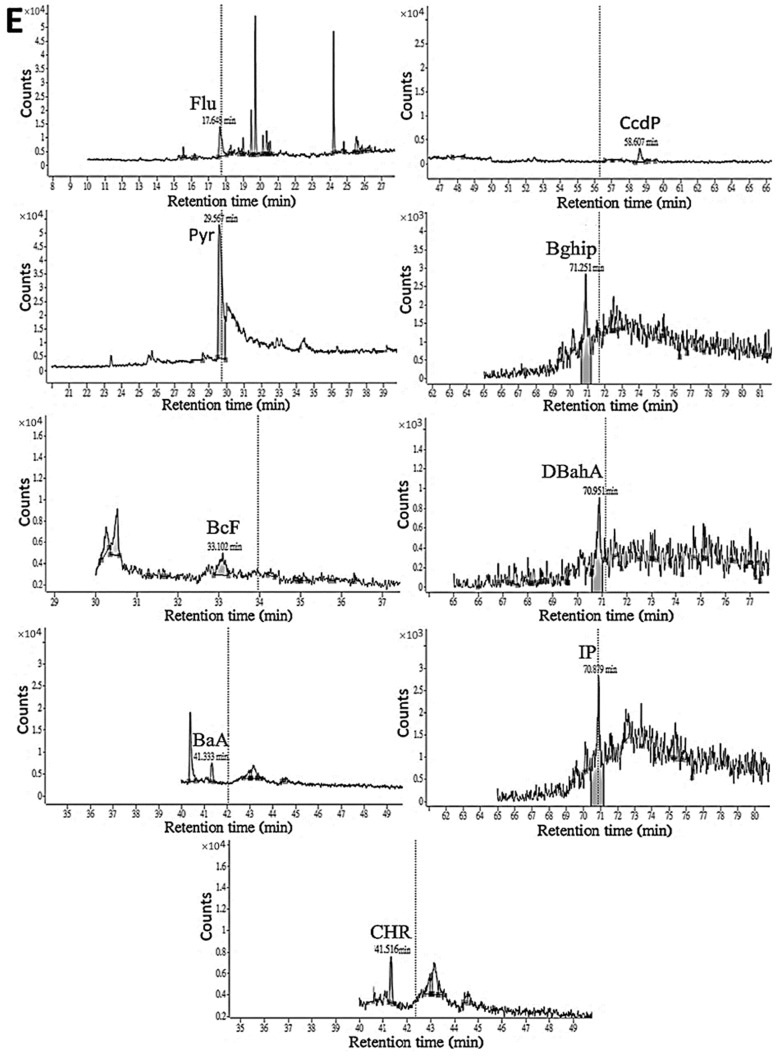

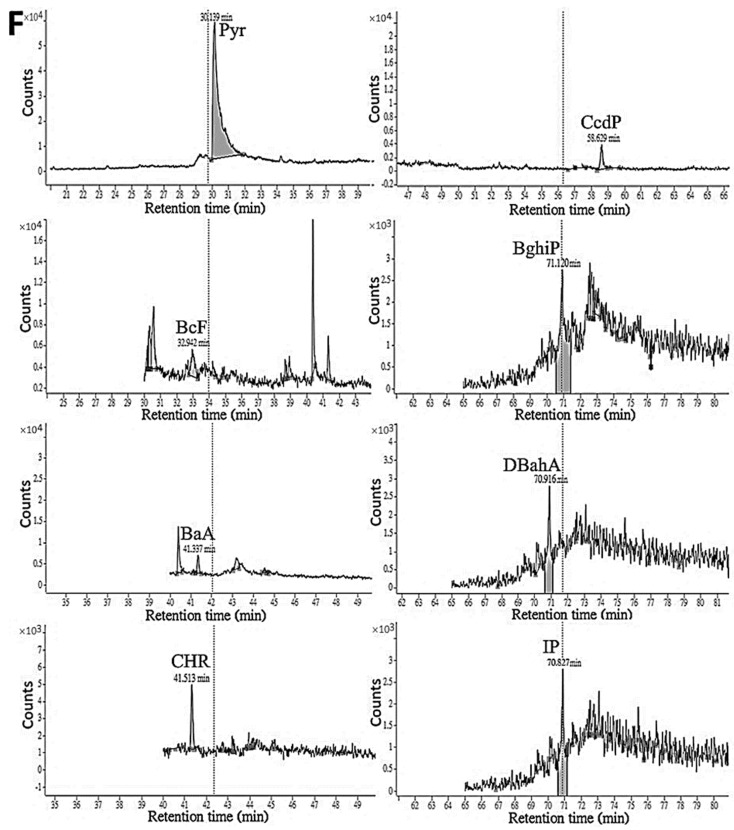

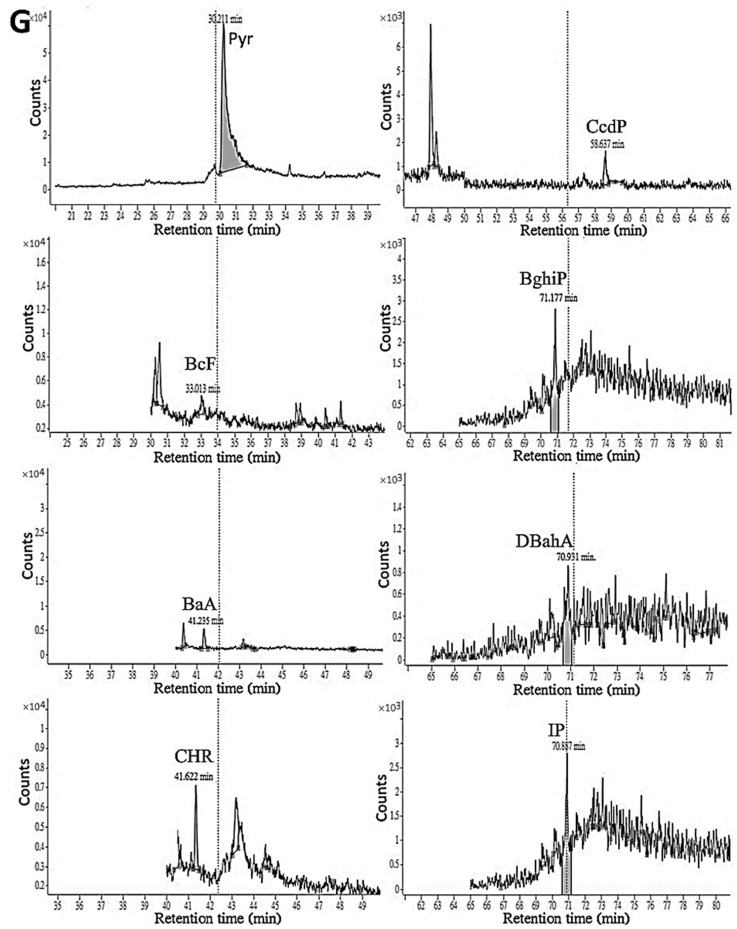
Chromatograms of PAH standards (**A**), as well as PAHs in raw pork (**B**) dried pork filaments (**C**), fried pork fiber (sesame oil, 160 °C/15 min) (**D**), fried pork fiber (sesame oil, 200 °C/6 min) (**E**), fried pork fiber (lard, 160 °C/15 min) (**F**), and fried pork fiber (lard, 200 °C/6 min) (**G**) in SRM mode via GC-MS/MS. The full names of the abbreviated individual PAHs are provided in Table S2.

Following the separation condition described in the Materials and Methods section, a total of 21 HA standards, along with an internal standard 4,7,8-TriMeIQx, were separated within 4 min (Figure 2A), while a total of 23 PAH standards, including internal standard Triphenylene, were separated within 78 min (Figure 3A). However, for PAH separation, both BbF and BjF overlapped.

### 3.2. Formation of HAs in Fried Pork Fiber as Affected by Oil Type and Processing Condition

Table 1 shows HA contents in raw pork, dried pork filament, and fried pork fiber as affected by oil type and processing condition. Only Harman (0.147 ng/g) was detected in raw pork (Figure 2B). However, following marination at 96 °C for 4 h and heating at 100 °C for 1.5 h, the Harman content rose to a much higher level (32.518 ng/g), accompanied by formation of Norharman (8.781 ng/g), DMIP (2.396 ng/g), IFP (0.641 ng/g), Trp-P-1 (0.099 ng/g), and Trp-P-2 (0.097 ng/g) in dried pork filament, with the total HAs being 44.532 ng/g (Figure 2C). Following frying at 160 °C/15 min or 200 °C/6 min with sesame oil, three more HA,s including MeIQ, PhIP, and MeAαC, were generated in fried pork fiber, with the total HAs being 142.721 ng/g for the former and 118.508 ng/g for the latter (Figure 2D,E). However, following frying at 160 °C/15 min or 200 °C/6 min with lard, four more HAs, including MeIQ, 8-MeIQx, PhIP, and MeAαC, were produced in fried pork fiber, with the total HAs being 91.852 ng/g for the former and 60.644 ng/g for the latter (Figure 2F,G). Comparatively, with sesame oil or lard as the frying medium, a greater content of total HAs was found in fried pork fiber processed at 160 °C for 15 min than at 200 °C for 6 min, implying that a longer heating time may play a more critical role in promoting HA formation than the heating temperature. Furthermore, by comparison at the same heating condition (160 °C/15 min or 200 °C/6 min), pork fiber fried in sesame oil produced a greater content of total HAs than that fried in lard, which can be attributed to the formation of a higher level of free fatty acid from sesame oil during frying. From a fatty acid composition point of view, sesame oil is more unsaturated than lard; thus, more unsaturated fatty acids such as linoleic acid were generated during stir-frying, leading to the formation of aldehyde-containing compounds for subsequent reaction with creatinine and pyridine or pyrazine for the formation of IQ or Iqx derivatives of HAs [16]. Alternatively, the aldehyde-containing compounds can react with tryptophan for the formation of 1,2,3,4-tetrahydrocarboline-3-carboxylic acid for further oxidation to generate Norharman [16]. Additionally, the main ingredient of marinated juice-soy sauce was shown to contain high levels of Harman and Norharman [17,18], which should also contribute to the formation of a large quantity of total HAs in fried pork fiber. In addition to soy sauce, roasting at a high temperature during processing of sesame seed oil was shown to promote the formation of Harman and Norharman [19].

For individual HAs, Harman was present in the highest amount in pork fiber fried with sesame oil, followed by Norharman, PhIP, IFP, DMIP, Trp-P-2, Trp-P-1, MeAαC, and MeIQ (Table 1). A similar tendency was shown for HA contents in pork fiber fried with lard, with the exception of PhIP, which was present in a much smaller amount, revealing that sesame oil was more important than lard in promoting PhIP formation. This result seems contradict a report by Pan et al. [20], who studied HA formation in meat floss (shredded and fried pork) as affected by three different types of edible oils. In their work, soybean oil was shown to possess the most pronounced effect in inhibiting HA formation, followed by lard and palm oil. It was postulated that the inhibition of HA formation in meat floss can be attributed to the presence of antioxidants such as vitamin E, β-carotene, and polyphenol compounds in soybean oil [21]. Conversely, the highly saturated lard was reported to accelerate HA formation through the liberation of more free fatty acids during heating [22]. Nevertheless, many studies have revealed that vegetable oils rich in polyunsaturated fatty acids such as linoleic acid should be more liable to HA formation, as the production of reactive oxygen species (ROS) from linoleic acid can induce the degradation of Amadori compounds to form Maillard reaction product intermediates 1-deoxysone and 3-dexoysone for subsequent HA generation [23,24]. In addition, Zamora et al. [25] also pointed out that the degradation products from lipid hydroperoxides during heating, such as methyl 13-hydroperoxyoctadeca-9,11-dienoate and alkenals, can also promote PhIP formation in a heated model system, while a greater amount of MeIQ and DiMeIQx was produced in fried beefburgers with a mixture of sunflower oil and margarine rich in oleic acid as the frying medium compared to butter and margarine [26]. On the contrary, a lower amount of MeIQx and DiMeIQx was generated in fried beefburgers with sunflower oil rich in linoleic acid as the frying medium, which may be due to the presence of a high amount of vitamin E [26].

By comparison, the pyrolytic HAs, such as Harman and Norharman, were the most susceptible to formation in fried pork fiber, which may be caused by amino acid degradation during stir-frying and soy sauce in marinated juice during heating. In a study dealing with formation of HAs in fried chicken fiber via different flavoring and processing condition, Hsu and Chen [17] also reported that both Harman and Norharman were the most dominant HAs generated during stir-frying at 150 °C/40 min or 180 °C/20 min with lard or soybean oil as the frying medium, while the other HAs, including Iqx, IFP, Trp-P-1, Phe-P-1, and PhIP, were present in small or trace amounts. This result is similar to our finding in the present study.

Collectively, both degree of oil unsaturation and method of oil processing should be taken into account when studying HA formation in meat products during processing as the latter can affect the type and amount of antioxidants present in edible oil used for frying.

### 3.3. Changes in Reducing Sugar, Amino Acid, Creatine, and Creatinine Contents in Raw Pork, Dried Pork Filament and Fried Pork Fiber as Affected by Oil Type and Processing Condition

Table 2 and Table 3, respectively, show changes in individual amino acid and total amino acid levels, and reducing sugar, creatine, and creatinine contents in raw pork, dried pork filament and fried pork fiber as affected by oil type and processing conditions. Compared to raw pork, the reducing sugar content rose by 4.08 mg/g in dried pork filament, which could be due to the conversion of sucrose to glucose and fructose during heating at 100 °C for 1.5 h. However, the reducing sugar contents declined to 1.98, 2.43, and 3.03 mg/g following stir-frying in sesame oil at 160 °C for 15 min and 200 °C for 6 min, as well as in lard at 160 °C for 15 min, respectively. This outcome revealed that reducing sugar participated in HA formation via reaction with amino acid and creatine/creatinine. Conversely, the reducing sugar rose by 4.30 mg/g following stir-frying in lard at 200 °C for 6 min, probably caused by the decomposition rate of sucrose being higher than the Maillard reaction (non-enzymatic browning) rate during stir-frying, resulting in a low amount of HAs formed in fried fish fiber.

Total amino acid content (Table 2 and Table 3) increased by 1.96 mg/g in dried pork filament following heating at 100 °C for 1.5 h, which can be attributed to a drop in moisture content during heating and the possible leaching of amino acids from soy sauce into pork during marination. However, only a minor change in total amino acid contents was found in pork fiber fried in sesame oil or lard at 160 °C/15 min and 200 °C/6 min. As amino acid is also a vital precursor for HA formation, this result indicated that a lesser amount of amino acids participated in HA formation via reaction with reducing sugar and creatine/creatinine [9]. It is also possible that the amino acid degradation may be responsible for formation of HAs such as Harman and Norharman in pork fiber during stir-frying. Additionally, like total amino acids, the individual amino acid content also showed a slight change during the processing of dried pork filament and fried pork fiber.

Table 3 also illustrates changes in creatine and creatinine contents in raw pork, dried pork filament, and fried pork fiber during processing. Only a low level of creatine (11.52 mg/100 g) was shown in raw pork, while a much greater content (73.75 mg/100 g) was found in dried pork filament, which should be caused by moisture loss and partial hydrolysis of creatine into creatinine during heating, as evidenced by a greater amount of creatinine (133.64 mg/100 g) present in dried pork filament. Following stir-drying in sesame oil or lard at 160 °C/15 min and 200 °C/6 min, the creatine contents declined while the creatinine contents increased for all the treatments, apparently due to the hydrolysis of creatine into creatinine, facilitating the Maillard reaction. Moreover, the creatine contents were greater in pork fiber processed at 200 °C/6 min than at 160 °C/15 min, indicating that a greater amount of creatine was converted to creatinine for the latter. Conversely, the creatinine content rose by 121.04 mg/100 g following heating at 100 °C for 1.5 h, which could be due to the conversion of creatine, an energy source usually present in creatine phosphate form in animal muscle tissue, for the subsequent reaction with amino acid/reducing sugar for HA formation. Additionally, the creatinine contents continued to rise during stir-frying and reached 154.14, 153.92, 146.19, and 149.65 mg/100 g in pork fiber fried in sesame oil at 160 °C/15 min and 200 °C/6 min and in lard at 160 °C/15 min and 200 °C/6 min, respectively. A comparison at the same heating conditions showed greater creatinine content in pork fiber fried in sesame oil than in that fried in lard. Apparently, during the stir-frying of pork fiber, the highly unsaturated sesame oil could favor the formation of creatinine compared to the more saturated lard, leading to a rise in total HA contents. As creatinine is a precursor of imidazole formation and can participate in the Maillard browning reaction, this outcome also implied that the formation rate of creatinine from creatine was higher than the Maillard reaction rate during stir-frying. An analogous result was observed for the creatine/creatinine content changes in ground pork and juice during marination at 90 °C for 24 h [10].

### 3.4. Formation of PAHs in Fried Pork Fiber as Affected by Oil Type and Processing Condition

Table 4 shows changes of PAH contents in dried pork filament and fried pork fiber as affected by oil type and processing condition. Only Pyr (8.727 ng/g), along with trace amounts of DbahA, DbaeP, and DbaiP, was detected in raw pork (Figure 3B); however, following marination and heating at 100 °C for 1.5 h, the Pyr content rose to 12.919 ng/g, while some PAHs, including BaA (3.299 ng/g), CHR (4.102 ng/g), CcdP (17.010 ng/g), and IP (3.134 ng/g), along with trace amounts of BcF, BbF, BjF, BghiP, DbahA, DbaeP, and DbaiP, were detected in dried pork filaments (Figure 3C). For the subsequent stir-frying of pork fiber in sesame oil or lard, most PAH contents demonstrated a slight change, with the exception of Pyr and CcdP, both of which showed a pronounced increase. Furthermore, one more PAH (Flu) was generated in pork fiber fried in sesame oil at 160 °C /15 min and 200 °C/6 min (Figure 3D,E). Comparatively, CcdP and Pyr were the most abundant PAHs generated in pork fiber fried in sesame oil or lard at 160 °C/15 min and 200 °C/6 min (Figure 3F,G). For total PAHs, the highest content (65.262 ng/g) was shown for pork fiber fried in lard at 200 °C /6 min, followed by frying in sesame oil at 200 °C/6 min (63.040 ng/g) and 160 °C/15 min (60.490 ng/g) and in lard at 160 °C/15 min (56.191 ng/g). By comparison, at the same heating condition (200 °C/6 min), a slight difference in total PAH contents was shown in pork fiber fried in lard and sesame oil. However, a greater content of total PAHs was generated in pork fiber fried in sesame oil than in lard at 160 °C/15 min, possibly caused by formation of a higher level of aldehyde-containing compounds from the former for subsequent PAH formation as sesame oil is rich in linoleic acid. Obviously, the compounds with benzene ring can act as potential precursors for PAH formation, as pointed out by Chen and Chen [27], who reported that PAHs can be formed by a reaction between benzaldehyde and 1,3-butadiene (formed from heat-induced linoleic acid oxidation), followed by a Diels–Alder cycloaddition reaction. Moreover, naphthalene can be formed from 4-pentyl-2,3-dimethyl-benzoic acid obtained from the reaction between trans,trans-2,4-decadienal (linoleic acid degradation product) and 2-butene (a dienophile) or from oxidation of 2-cyclohexene-1-one for the subsequent reaction with C4-containing compounds via Diels–Alder reaction [9].

The effect of oil type on PAH formation has been controversial. For instance, Zhu and Wang [28] reported that at the same heating temperature, lard was the most susceptible to PAH formation in the smoke, followed by soybean oil and rapeseed oil. However, with a model system, following a rise in unsaturation of lipid precursors such as methyl stearate, methyl oleate, methyl linoleate, and methyl linolenate, the PAH contents also increased [29]. Similarly, polyunsaturated fatty acids were reported to be the most susceptible to PAH formation, followed by monounsaturated fatty acids and saturated fatty acids, in a model system during heating, with the high-level PAHs with four or five rings being generated from polyunsaturated fatty acids at elevated temperatures up to 240 °C [30]. However, this phenomenon may vary with meat products during processing. For example, Liu et al. [31] studied the effect of fatty acids on PAH formation in chicken products during roasting and reported that the greater the degree of unsaturation of fatty acids, the greater the formation of PAHs. However, in a similar study dealing with the effect of vegetable oil as fat replacer on PAH formation in pork patties, grapeseed oil (replaced 40% fat) was shown to promote the formation of total PAHs and BaP, while olive oil reduced the formation of total PAHs and BaP [21]. However, a different outcome was found for sunflower oil, as it was effective in reducing BaP formation at 180 °C but promoting BaP formation at 220 °C [21]. On the basis of fatty acid composition, both grapeseed oil and sunflower oil are rich in polyunsaturated fatty acids and should be more liable to PAH formation than olive oil. Thus, the presence of high levels of antioxidants such as α-tocopherol in sunflower oil could be responsible for BaP inhibition at 180 °C, while at 220 °C, α-tocopherol can undergo degradation and thus fails to inhibit PAH formation.

### 3.5. Changes of PAH Precursor Contents in Fried Pork Fiber as Affected by Oil Type and Processing Condition

Table 5 shows content changes of PAH precursors, including 4,4-dimethyl-2-cyclohexene-1-one, 2-cyclohexene-1-one, cyclohexene, benzaldehyde, and trans,trans-2,4-decadienal, in fried pork fiber as affected by oil type and processing condition. Only 2-cyclohexene (5.75 ng/g) and trans,trans-2,4-decadienal (7.28 ng/g) were detected in dried pork filament, while 4,4-dimethyl-2-cyclohexene-1-one, cyclohexene, and benzaldehyde remained undetected. However, following the stir-frying of pork fiber in sesame oil at 160 °C/15 min and 200 °C/6 min, the 2-cyclohexene-1-one content rose, respectively, to 7.68 and 8.73 ng/g, while the trans,trans-2,4-decadienal level increased to 14.73 and 31.50 ng/g, with the concomitant formation of a high level of benzaldehyde equaling 288.62 and 365.76 ng/g, respectively. A similar trend was shown for pork fiber fried in lard at 160 °C/15 min and 200 °C/6 min, as evident by a rise of the 2-cyclohexene-1-one content to 9.91 and 70.74 ng/g and the trans,trans-2,4-decadienal level to 26.56 and 213.26 ng/g, with the concomitant generation of a high level of benzaldehyde, amounting to 193.93 and 404.00 ng/g, respectively.

Comparatively, benzaldehyde was generated in greater amounts than 2-cyclohexene-1-one and trans,trans-2,4-decadienal in fried pork fiber, and it should play a more critical role in PAH formation as it was inferred that benzaldehyde could be produced through cyclohexene oxidation, while 2-cyclohexene-1-one and trans,trans,2,4-decadienal were generated through the degradation of linolenic acid and linoleic acid, respectively, during heating [27]. Additionally, the major ingredient of marinated juice, namely, sugar, may contribute to PAH formation, as 5-HMF, methylglyoxal and acetone compounds formed upon dehydration of sugar under acidic condition can lead to subsequent indene formation from 5-HMF via Diels–Alder reaction and dehydrogenation and acetylene addition, resulting in the formation of PAHs (FL) [10]. Furthermore, the reducing sugar glucose formed from sucrose during heating was shown to be more liable to PAH formation than fructose, which may be due to the generation of small aldehyde compounds from the former for the eventual condensation or polymerization to generate PAHs [32]. Nevertheless, a reaction between sugar and amino acid during heating may produce a product with sufficient antioxidant activity for PAH inhibition [9]. Thus, highly toxic PAHs such as BaP remained undetected in fried pork fiber for all the processing treatments, which can be ascribed to the formation of Maillard reaction products possessing antioxidant activity [9,33]. Besides sugar, soy sauce was also reported to contain antioxidants such as isoflavone at 8.17 μg/mL [34]. Therefore, the addition of soy sauce and sugar during marination may also inhibit PAH formation in pork fiber during stir-frying. Interestingly, in addition to antioxidant activity, the Maillard reaction products may also exert pro-oxidant activity depending on the type and level of products formed during heating [9]. Moreover, under the same heating conditions (160 °C, 15 min), the PAH precursor contents in pork fiber fried in sesame oil were greater than in lard. As explained above, sesame oil containing a much higher level of linoleic acid than lard should be more susceptible to the formation of 2-cyclohexene-1-one, benzaldehyde, and trans,trans-2,4-decadienal in pork fiber during stir-frying. Nevertheless, we have to point out that both precursors—4,4-dimethyl-2-cyclohexene-1-one and cyclohexene—remained undetected for all the processing treatments. Taken together, the oil type, flavoring material, and processing condition could play a critical role in PAH formation or inhibition in pork fiber during stir-frying.

For toxicity comparison of HAs and PAHs (trace amount not included), a total of five possible human carcinogens (HAs), including MeIQ, 8-MeIQx, Trp-P-1, Trp-P-2, and PhIP, were detected in pork fiber fried in lard, while four possible human carcinogens (HAs), including Trp-P-1, Trp-P-2, PhIP, and MeAαC, were present in pork fiber fried in sesame oil. For PAHs, a total of three probable human carcinogens, including CcdP, DbahA, and DbalP, and seven possible human carcinogens, including BaA, CHR, MCH, BbF, BjF, DbaiP, and IP, were detected in pork fiber fried in lard, while two probable human carcinogens, CcdP and DbahA, and the same seven possible human carcinogens as mentioned above were present in pork fiber fried in sesame oil. Although one more probable human carcinogen (DbalP) was produced in pork fiber fried in lard than in sesame oil, the highly toxic BaP remained undetected in fried pork fiber for all the treatments. Thus, from a safety perspective, the health risk associated with consumption of fried pork fiber should be minimal.

### 3.6. Factorial Design Analysis

A two-factorial analysis of HAs and PAHs formation as affected by oil type and frying condition was performed using the two-way ANOVA method, and the results are shown in Table 6. A *p*-value of < 0.0001 was shown for both frying condition and oil type, indicating that these two factors individually possessed a significant impact on HA formation in fried pork fiber. However, the interaction between frying condition and oil type was insignificant as a *p*-value of 0.2336 was obtained. This observation is in accordance with the foregoing discussion of the results shown in Table 1, implying that HAs were more susceptible to formation by the low-temperature long-time condition (160 °C/15 min) than by the high-temperature short-time condition (200 °C/6 min) with the same oil type. However, under the same frying condition, the addition of sesame oil can better facilitate HA formation compared to lard; on the other hand, the interaction between frying condition and oil type showed a significant impact on PAH formation (*p*-value, 0.0002) in fried pork fiber, while the frying condition (*p*-value, <0.0001) can more significantly promote PAH formation than the oil type (*p*-value, 0.1453). This outcome is in agreement with the foregoing discussion of the results shown in Table 4, suggesting that PAH was more prone to formation in high-temperature short-time (200 °C/6 min) conditions than that in low-temperature long-time (160 °C/15 min) conditions with the same oil type. However, the oil type showed less impact on PAH formation in pork fiber, as evidenced by the lower level of total PAH with lard (160 °C/15 min) or sesame oil (200 °C/6 min) as the frying medium under the same frying conditions.

### 3.7. Principal Component Analysis

**Figure 4:**
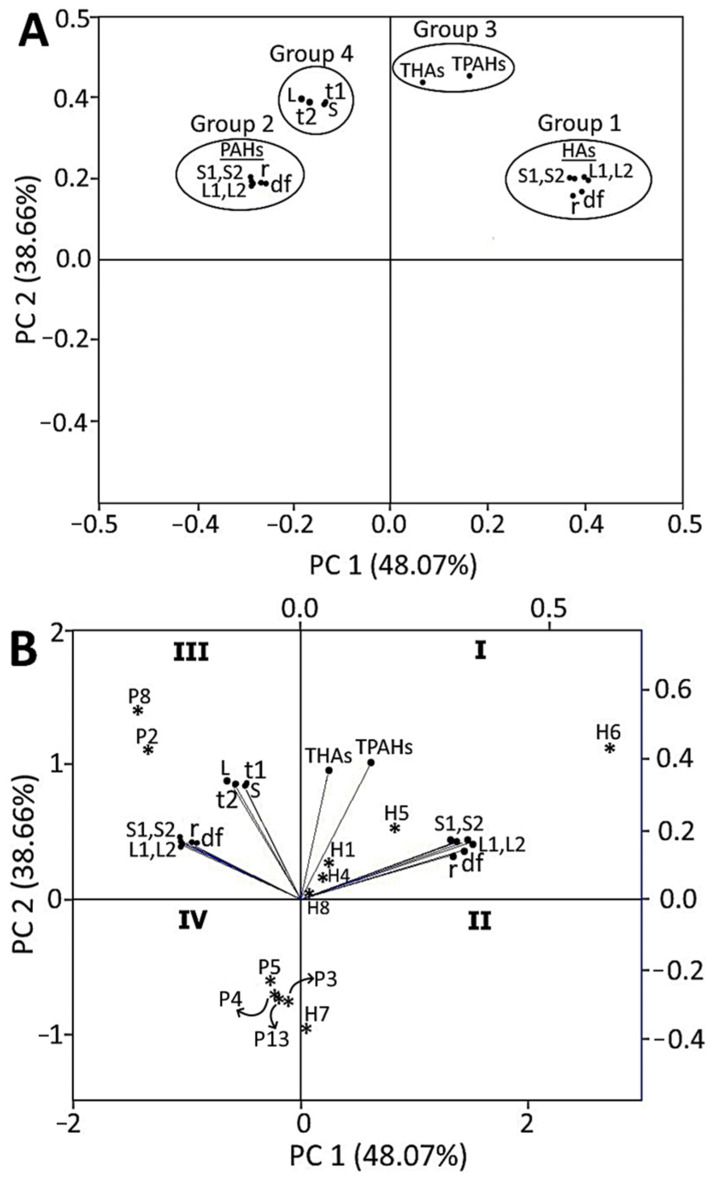
Principal component analysis showing score plot (**A**) and biplot containing loading plot and score plot (**B**) for HAs and PAHs formation as affected by oil type and processing conditions. HAs, heterocyclic amines; PAHs, polycyclic aromatic hydrocarbons; r, raw pork tenderloin; df, dried pork filament obtained by cutting pork tenderloin into pieces and marinating with juice containing 200 g of soy sauce (10%), 20 g of crystal sugar (1%) and 1780 g of distilled water (89%), for 4 h at 96 °C, followed by cooling, smashing with a wooden mallet to produce thread-like pork filament and drying in a pan at 100 °C for 1.5 h; S1, pork fiber stir-fried at 160 °C for 15 min with sesame oil added 5 min before the end of stir-frying; S2, pork fiber stir-fried at 200 °C for 6 min with sesame oil added 5 min before the end of stir-frying; L1, pork fiber stir-fried at 160 °C for 15 min with lard added 3 min before the end of stir-frying; L2, pork fiber stir-fried at 200 °C for 6 min with lard added 3 min before the end of stir-frying; S, the amount of HAs and PAHs formed regardless of temperature during stir-frying of pork fiber in sesame oil; L, the amount of HAs and PAHs formed regardless of temperature during stir-frying of pork fiber in lard; t1, the amount of HAs and PAHs formed regardless of oil type during stir-frying of pork fiber at 160 °C for 15 min; t2, the amount of HAs and PAHs formed regardless of oil type during stir-frying of pork fiber at 200 °C for 6 min. I, II, III and IV in (**B**) represent quadrant I, II, III and IV, respectively. The asterisk (*) in (**B**) denotes the six highly formed individual HA or PAH compounds as affected by different oil types and processing conditions. H1, DMIP; H4, IFP; H5, Norharman; H6, Harman; H7, Trp-P-2; H8, PhIP; P2, Pyr; P3, BcF; P4, BaA; P5, CHR; P8, CcdP; P13, IP. Mean data of triplicate determinations of HAs or PAHs were used for PCA analysis.

The PCA for formation of HAs and PAHs as affected by different oil types and processing conditions is shown in Figure 4. Based on an eigen value of the correlation matrix > 1, a total of three components (PCs 1–3) were shown to describe all the treatment conditions, of which the first two components, PC 1 (48.07%) and PC 2 (38.66%), accounted for the maximum total variation of 86.73% in HAs and PAHs formation as affected by different oil types and processing conditions. The score plot in Figure 4A shows that the experimental mean data can be grouped into four groups based on the proximity of data points and the extent of the separation of one group from the other, with Group 1 and Group 2 denoting various individual HAs and PAHs formed, respectively, with six different treatments, including raw pork tenderloin (r), dried pork filament (marinated at 96 °C for 4 h and dried at 100 °C for 1.5 h) (df), sesame oil-stir-fried pork fiber at 160 °C for 15 min (S1), sesame oil-stir-fried pork fiber 200 °C for 6 min (S2), lard-stir-fried pork fiber at 160 °C for 15 min (L1), and lard-stir-fried pork fiber at 200 °C for 6 min (L2). Evidently, the separation of Group 1, representing the HA formation for all the six treatments, and Group 2, representing PAH formation, was distinct, signifying that HAs and PAHs are formed via different mechanisms. Apparently, the analysis of contents of HAs and PAHs precursors in this study revealed that the reaction of amino acids from soy sauce and dried pork filament with reducing sugar and creatine/creatinine generated HAs in pork fiber during stir-frying in sesame oil or lard, while benzaldehyde, 2-cyclohexene-1-one, and trans,trans-2,4-decadienal were responsible for PAHs formation during stir-frying. On the other hand, regardless of oil type and processing condition, the total contents of HAs (THAs) and PAHs (TPAHs) formed during different treatments were grouped as Group 3, indicating that both were formed in significant amounts at all studied conditions, as shown, respectively, in the range of 0.147–142.721 ng/g and 8.727–65.262 ng/g in Table 1 and Table 4. Similarly, the total contents of HAs and PAHs formed in sesame oil (S) or lard (L), regardless of temperature, as well as the total contents of HAs and PAHs formed in stir-fried pork fiber at 160 °C for 15 min (t1) and 200 °C for 6 min (t2), regardless of oil type, are grouped as Group 4, confirming again that both HAs and PAHs are formed to significant degrees in all the treatment conditions studied. Relatively, the score plots in Group 1 were more scattered compared to that in Group 2, implying that HAs are more liable to formation than PAHs under the studied processing conditions.

Figure 4B illustrates the biplot containing both loading plots and score plots, with a larger angle between the loading plots shown for Group 1 and Group 2, as well as a significant angle between total HA and PAH contents in Group 3 (THAs and TPAHs), further corroborating that HAs and PAHs are formed via different mechanisms. Moreover, a significant angle was observed between the loading plots of total HAs and PAHs formed in sesame oil- and lard-stir-fried pork fiber (S and L), as well as that processed at 160 °C for 15 min and 200 °C for 6 min (t1 and t2) in Group 4, revealing that the formation of both HAs and PAHs was significantly affected by the difference in oil type and stir-frying temperature. Furthermore, the inclination of loading plots for HA formation in raw pork, dried pork filament, and sesame oil- or lard-stir-fried pork fiber at 160 °C and 200 °C towards the PC 1 direction (48.07%), and that of PAH formation towards the PC 2 direction (38.66%), demonstrated their corresponding impact on PC 1 and PC 2, with HAs being more prone to formation than PAHs as their loading plots are inclined towards the higher percentage component PC 1 (48.07%).

The score plots designated by asterisks in Figure 4B denote the individual HA or PAH compound formation as affected by oil type and processing condition. The presence of six asterisks that represent six individual HA compounds in quadrant I and II showed that their formation, regardless of oil type and processing condition, highly influenced the PC 1 in the following order: Harman (H6) > Norharman (H5) > DMIP (H1) > IFP (H4) > PhIP (H8) > Trp-P-2 (H7), as the asterisk position moves towards the vertical zero line in the middle of Figure 4B. Similarly, the presence of six asterisks that represent six individual PAH compounds in quadrant III and IV showed that their formation, regardless of oil type and processing condition, highly impacted the PC 2 in the following order: CcdP (P8) > Pyr (P2) > CHR (P5) > BaA (P4) > IP (P13) > BcF (P3). In addition, the proximity of asterisks denoting HA compounds Harman, Norharman, DMIP, IFP and PhIP as well as those denoting PAH compounds CcdP and Pyr on the positive quadrants I and III of PC1 and PC2, respectively, also suggested their highest formation when compared to other HA or PAH compounds. These observed trends correlated well with the row-wise total contents for each HA or PAH, regardless of oil type and processing condition, as shown in Table 1 and Table 4. Thus, the biplot in Figure 4B, containing both loading plots and score plots, illustrates a complete grouping and correlation for HA or PAH formation as affected by oil type and processing condition. Collectively, the PCA revealed a significant formation of both HAs and PAHs in stir-fried pork fiber through different mechanisms, with the former being more prone to formation, as evident by Harman and Norharman dominating in HA formation, while CcdP and Pyr dominate in PAH formation.

## 4. Conclusions

In conclusion, the various HAs and PAHs formed in pork fiber during stir-frying were studied. A greater amount of total HAs was generated in pork fiber fried at 160 °C for 15 min than at 200 °C for 6 min with either sesame oil or lard as the frying medium. However, in the same heating conditions, a greater content of total HAs and total PAHs was produced in pork fiber fried in sesame oil than in lard. Moreover, the greatest amount of total PAHs was found in pork fiber fried in lard at 200 °C/6 min, followed by that fried in sesame oil at 200 °C/6 min and 160 °C/15 min, and in lard at 160 °C/15 min. The HA and PAH precursors were also determined in order to postulate the formation mechanism in fried pork fiber. PCA analysis also demonstrated the different formation mechanisms of HAs and PAHs in fried pork fiber.

## Figures and Tables

**Table 1:** HA contents (ng/g) in raw pork, dried pork filament, and fried pork fiber as affected by oil type and processing condition ^1,2^.

HA ^3^	Raw Pork	Dried Pork Filament	Fried Pork Fiber			
			Sesame Oil (160 °C/15 min)	Sesame Oil (200 °C/6 min)	Lard (160 °C/15 min)	Lard (200 °C/6 min)
DMIP	nd ^4^	2.396 ± 0.21 ^d^	8.564 ± 0.457 ^b^	6.615 ± 1.254 ^c^	12.636 ± 1.187 ^a^	5.602 ± 0.334 ^c^
MeIQ	nd	nd	trace ^5^	trace	0.684 ± 0.043 ^a^	0.398 ± 0.089 ^b^
8-MeIQx	nd	nd	nd	nd	0.372 ± 0.064 ^a^	trace
IFP	nd	0.641 ± 0.051 ^d^	10.297 ± 1.150 ^a^	8.182 ± 0.171 ^b^	7.216 ± 0.488 ^b^	4.224 ± 0.135 ^c^
Norharman	trace	8.781 ± 0.249 ^d^	45.959 ± 2.278 ^a^	36.104 ± 1.503 ^b^	18.292 ± 1.056 ^c^	10.113 ± 0.681 ^d^
Harman	0.147 ± 0.014 ^e^	32.518 ± 2.924 ^d^	64.311 ± 3.898 ^a^	55.768 ± 3.928 ^b^	50.477 ± 0.481 ^b^	40.065 ± 1.792 ^c^
Trp-P-2	nd	0.097 ± 0.030 ^d^	2.021 ± 0.065 ^a^	1.891 ± 0.081 ^b^	1.374 ± 0.067 ^c^	0.120 ± 0.015 ^d^
PhIP	nd	nd	11.108 ± 0.966 ^a^	9.439 ± 0.411 ^b^	0.655 ± 0.047 ^c^	trace
Trp-P-1	trace	0.099 ± 0.063 ^b^	0.322 ± 0.076 ^a^	0.361 ± 0.101 ^a^	0.145 ± 0.085 ^b^	0.122 ± 0.077 ^b^
MeAαC	nd	nd	0.138 ± 0.013 ^a^	0.147 ± 0.009 ^a^	trace	trace
Total	0.147 ± 0.014 ^f^	44.532 ± 3.129 ^e^	142.721 ± 6.947 ^a^	118.508 ± 5.601 ^b^	91.852 ± 2.358 ^c^	60.644 ± 2.880 ^d^

^1^ Data are presented as mean ± standard deviation of triplicate determinations. ^2^ Data with different superscripts (a–f) in the same row are significantly different at α = 0.05 level. ^3^ The full name of individual HA is shown in Table S1. ^4^ not detected. ^5^ LOQ ≥ HAs levels ≥ LOD.

**Table 2:** Amino acid contents in raw pork, dried pork filament, and fried pork fiber as affected by oil type and processing condition ^1,2^.

Amino Acid (mg/g)	Raw Pork	Dried Pork Filament	Fried Pork Fiber			
			Sesame Oil	Sesame Oil	Lard	Lard
			(160 °C/15 min)	(200 °C/6 min)	(160 °C/15 min)	(200 °C/6 min)
Aspartic acid	0.56 ± 0.04 ^b^	0.73 ± 0.04 ^a^	0.65 ± 0.05 ^ab^	0.73 ± 0.05 ^a^	0.57 ± 0.04 ^b^	0.68 ± 0.14 ^ab^
Glutamic acid	1.00 ± 0.05 ^c^	1.55 ± 0.11 ^a^	1.47 ± 0.06 ^ab^	1.62 ± 0.07 ^a^	1.29 ± 0.09 ^b^	1.51 ± 0.23 ^a^
Serine	0.24 ± 0.01 ^c^	0.45 ± 0.05 ^ab^	0.47 ± 0.03 ^ab^	0.50 ± 0.01 ^a^	0.41 ± 0.03 ^b^	0.51 ± 0.07 ^a^
Histidine	0.07 ± 0.03 ^b^	0.09 ± 0.01 ^ab^	0.06 ± 0.01 ^b^	0.08 ± 0.01 ^b^	0.12 ± 0.05 ^a^	0.10 ± 0.01 ^ab^
Glycine	0.46 ± 0.01 ^a^	0.44 ± 0.10 ^a^	0.39 ± 0.06 ^a^	0.46 ± 0.03 ^a^	0.36 ± 0.15 ^a^	0.51 ± 0.15 ^a^
Threonine	0.13 ± 0.01 ^c^	0.24 ± 0.02 ^ab^	0.23 ± 0.01 ^ab^	0.26 ± 0.01 ^ab^	0.22 ± 0.01 ^b^	0.26 ± 0.05 ^a^
Arginine	0.65 ± 0.01 ^c^	0.81 ± 0.09 ^ab^	0.84 ± 0.03 ^ab^	0.89 ± 0.02 ^a^	0.77 ± 0.04 ^b^	0.80 ± 0.02 ^b^
Alanine	0.52 ± 0.02 ^c^	0.59 ± 0.04 ^bc^	0.60 ± 0.02 ^bc^	0.63 ± 0.02 ^ab^	0.50 ± 0.05 ^c^	0.69 ± 0.10 ^a^
Tyrosine	0.22 ± 0.01 ^c^	0.31 ± 0.04 ^ab^	0.28 ± 0.01 ^ab^	0.32 ± 0.01 ^ab^	0.29 ± 0.01 ^b^	0.32 ± 0.03 ^a^
Cystine	0.05 ± 0.00 ^a^	0.06 ± 0.00 ^a^	0.05 ± 0.01 ^a^	0.07 ± 0.02 ^a^	0.06 ± 0.01 ^a^	0.07 ± 0.02 ^a^
Valine	0.32 ± 0.01 ^d^	0.44 ± 0.03 ^ab^	0.42 ± 0.02 ^bc^	0.46 ± 0.03 ^ab^	0.37 ± 0.02 ^cd^	0.50 ± 0.08 ^a^
Methionine	0.21 ± 0.01 ^a^	0.27 ± 0.02 ^a^	0.28 ± 0.04 ^a^	0.29 ± 0.02 ^a^	0.25 ± 0.03 ^a^	0.25 ± 0.01 ^a^
Phenylalanine	0.25 ± 0.01 ^c^	0.54 ± 0.06 ^ab^	0.54 ± 0.01 ^ab^	0.57 ± 0.02 ^a^	0.49 ± 0.02 ^b^	0.55 ± 0.04 ^a^
Isoleucine	0.31 ± 0.01 ^c^	0.42 ± 0.03 ^a^	0.41 ± 0.02 ^ab^	0.44 ± 0.02 ^a^	0.35 ± 0.03 ^bc^	0.43 ± 0.07 ^a^
Leucine	0.67 ± 0.02 ^c^	0.98 ± 0.09 ^a^	1.03 ± 0.03 ^a^	1.08 ± 0.04 ^a^	0.86 ± 0.08 ^b^	1.01 ± 0.09 ^a^
Lysine	0.78 ± 0.16 ^a^	0.42 ± 0.20 ^b^	0.31 ± 0.08 ^b^	0.37 ± 0.05 ^b^	0.32 ± 0.06 ^b^	0.34 ± 0.20 ^b^
Proline	0.38 ± 0.07 ^b^	0.44 ± 0.07 ^b^	0.44 ± 0.03 ^b^	0.80 ± 0.42 ^a^	0.28 ± 0.16 ^b^	0.40 ± 0.01 ^b^
Total amino acid (mg/g)	6.82 ± 0.16 ^c^	8.78 ± 0.58 ^a^	8.49 ± 0.31 ^ab^	9.58 ± 0.51 ^a^	7.50 ± 0.73 ^bc^	8.92 ± 1.26 ^a^

^1^ Data are presented as mean of triplicate analyses ± standard deviation. ^2^ Data with different superscripts (a–d) in the same row are significantly different at α = 0.05 level.

**Table 3:** Reducing sugar, amino acid, creatine, and creatinine contents in raw pork, dried pork filament, and fried pork fiber as affected by oil type and processing condition ^1,2^.

	Raw Pork	Dried Pork Filament	Fried Pork Fiber			
			Sesame Oil	Sesame Oil	Lard	Lard
			(160 °C/15 min)	(200 °C/6 min)	(160 °C/15 min)	(200 °C/6 min)
Reducing sugar (mg/g)	nd ^3^	4.08 ± 0.07 ^b^	1.98 ± 0.09 ^e^	2.43 ± 0.23 ^d^	3.03 ± 0.07 ^c^	8.38 ± 0.18 ^a^
Amino acid (mg/g)	6.82 ± 0.16 ^c^	8.78 ± 0.58 ^a^	8.49 ± 0.31 ^ab^	9.58 ± 0.51 ^a^	7.50 ± 0.73 ^bc^	8.92 ± 1.26 ^a^
Creatine (mg/100 g)	11.52 ± 0.28 ^f^	73.75 ± 1.92 ^a^	29.12 ± 0.22 ^d^	37.89 ± 0.97 ^c^	25.77 ± 0.93 ^e^	47.75 ± 0.06 ^b^
Creatinine (mg/100 g)	12.60 ± 0.28 ^d^	133.64 ± 1.16 ^c^	154.14 ± 0.95 ^a^	153.92 ± 5.18 ^a^	146.19 ± 0.53 ^b^	149.65 ± 0.50 ^ab^

^1^ Data are presented as mean ± standard deviation of triplicate determinations. ^2^ Data with different superscripts (a–f) in the same row are significantly different at α = 0.05 level. ^3^ not detected.

**Table 4:** PAH contents (ng/g) in raw pork, dried pork filament, and fried pork fiber as affected by oil type and processing condition ^1,2^.

PAH ^3^	Raw Pork	Dried Pork Filament	Fried Pork Fiber			
			Sesame Oil (160 °C/15 min)	Sesame Oil (200 °C/6 min)	Lard (160 °C/15 min)	Lard (200 °C/6 min)
Flu	nd ^4^	nd	0.119 ± 0.101 ^a^	0.177 ± 0.090 ^a^	nd	nd
Pyr	8.727 ± 0.069 ^d^	12.919 ± 0.020 ^c^	17.980 ± 0.018 ^b^	19.627 ± 0.497 ^ab^	17.893 ± 0.021 ^b^	20.906 ± 0.046 ^a^
BcF	nd	trace ^5^	3.486 ± 0.278 ^a^	2.965 ± 0.074 ^b^	2.587 ± 0.020 ^c^	2.636 ± 0.011 ^c^
BaA	nd	3.299 ± 0.004 ^ab^	3.324 ± 0.008 ^a^	3.259 ± 0.019 ^bc^	3.252 ± 0.006 ^c^	3.315 ± 0.048 ^a^
CHR	nd	4.102 ± 0.004 ^a^	4.126 ± 0.008 ^a^	4.070 ± 0.022 ^a^	4.056 ± 0.005 ^a^	3.578 ± 0.941 ^a^
BbF	nd	trace	trace	trace	trace	trace
BjF	nd	trace	trace	trace	trace	trace
CcdP	nd	17.010 ± 0.095 ^e^	24.400 ± 0.605 ^c^	25.854 ± 0.494 ^b^	22.030 ± 0.256 ^d^	27.527 ± 0.423 ^a^
BghiP	nd	trace	1.649 ± 0.092 ^a^	1.603 ± 0.059 ^a^	1.379 ± 0.049 ^a^	1.964 ± 0.984 ^a^
DBahA	trace	trace	2.070 ± 0.029 ^b^	2.196 ± 0.043 ^a^	1.902 ± 0.009 ^c^	1.901 ± 0.018 ^c^
DBaeP	trace	trace	trace	trace	trace	trace
DBaiP	trace	trace	trace	trace	trace	trace
IP	nd	3.134 ± 0.017 ^a^	3.337 ± 0.041 ^a^	3.288 ± 0.033 ^a^	3.092 ± 0.020 ^a^	3.433 ± 0.560 ^a^
Total	8.727 ± 0.069 ^f^	40.464 ± 0.11 ^e^	60.490 ± 0.370 ^c^	63.040 ± 1.780 ^b^	56.191 ± 0.299 ^d^	65.262 ± 0.254 ^a^

^1^ Data are presented as mean ± standard deviation of triplicate determinations. ^2^ Data with different superscripts (a–f) in the same row are significantly different at α = 0.05 level. ^3^ The full name of individual PAH is shown in Table S2. ^4^ not detected. ^5^ LOQ ≥ HAs levels ≥ LOD.

**Table 5:** PAH precursor contents (ng/g) in raw pork, dried pork filament, and fried pork fiber as affected by oil type and processing condition.

Sample	Precursor Content (ng/g) ^1,2^					
	4,4-Dimethyl-2-Cyclohexene-1-One	2-Cyclohexene-1-One	Cyclohexene	Benzaldehyde	Trans,Trans-2,4-Decadienal	Total (ng/g)
Raw pork	nd ^3^	0.94 ± 0.12 ^c^	nd	nd	nd	0.94 ± 0.12 ^f^
Dried pork filament	nd	5.75 ± 0.80 ^b^	nd	nd	7.28 ± 0.73 ^d^	13.03 ± 1.54 ^e^
Fried pork fiber with sesame oil (160 °C/15 min)	nd	7.68 ± 0.97 ^b^	nd	288.62 ± 16.25 ^b^	14.73 ± 2.10 ^cd^	311.04 ± 18.66 ^c^
Fried pork fiber with sesame oil (200 °C/6 min)	nd	8.73 ± 0.11 ^b^	nd	365.76 ± 15.91 ^a^	31.50 ± 6.33 ^b^	405.99 ± 13.66 ^b^
Fried pork fiber with lard (160 °C/15 min)	nd	9.91 ± 1.14 ^b^	nd	193.93 ± 13.75 ^c^	26.56 ± 5.32 ^bc^	230.40 ± 19.81 ^d^
Fried pork fiber with lard (200 °C/6 min)	nd	70.74 ± 10.72 ^a^	nd	404.00 ± 31.44 ^a^	213.26 ± 18.56 ^a^	688.00 ± 47.79 ^a^

^1^ Data are presented as mean ± standard deviation of triplicate determinations. ^2^ Data with different superscripts (a–f) in the same column are significantly different at α = 0.05 level. ^3^ not detected.

**Table 6:** A two-factorial analysis of HAs and PAHs formation in pork fiber as affected by oil type and frying condition.

Factor	DF ^1^	SS ^2^	MS ^3^	F-Value	*p*-Value
HAs					
Frying condition	1	2303.59	2303.59	104.19	<0.0001
Oil type	1	8867.31	8867.31	401.05	<0.0001
Frying condition × Oil type	1	36.70	36.70	1.66	0.2336
PAHs					
Frying condition	1	107.15	107.15	122.29	<0.0001
Oil type	1	2.28	2.28	2.60	0.1453
Frying condition × Oil type	1	35.24	35.24	40.21	0.0002

^1^ degree of freedom. ^2^ sum of squares. ^3^ mean squares.

## Data Availability

The data that support the findings of this study are available within the manuscript.

## Supplementary Materials

The following supporting information can be downloaded at https://www.mdpi.com/article/10.3390/foods12183504/s1. Various materials and reagents used in this study are provided as text. In addition, Table S1 showing retention time and selected reaction monitoring (SRM) detection parameters of 20 HAs and internal standard (4,7,8-TriMeIQx) by UPLC-MS/MS and Table S2 showing retention time and selected reaction monitoring (SRM) detection parameters of 23 PAHs and internal standard (Triphenylene) by GC-MS/MS.
