# Supplementary material for: Effects of Oil and Processing Conditions on Formation of Heterocyclic Amines and Polycyclic Aromatic Hydrocarbons in Pork Fiber
Source: Foods. 2023 Sep 20;12(18):3504. doi: 10.3390/foods12183504 (PMC10528247; doi:10.3390/foods12183504)
Supplement: Supplementary file 1 [file foods-12-03504-s001.zip › foods-2569490-supplementary.pdf]

Supplementary material for

Effects of Oil and Processing Conditions on Formation of Heterocyclic Amines and Polycyclic Aromatic Hydrocarbons in Pork Fiber

Yu-Wen Lai[1], Baskaran Stephen Inbaraj[1], Bing-Huei Chen[1,2*]

[1]Department of Food Science, Fu Jen Catholic University, New Taipei City 242062, Taiwan.

[2]Department of Nutrition, China Medical University, Taichung 40402, Taiwan.

[*]To whom correspondence should be addressed

Email address: 002622@mail.fju.edu.tw

Phone: +886-2-29053626

Materials and methods

Materials and reagents

The raw pork tenderloin (1 kg) was procured from a local supermarket located in Xinzhuang District (New Taipei City, Taiwan). The standards of 24 PAHs as listed in Table S2 were purchased from Sigma-Aldrich Co (St. Louis, MO, USA), while the standards of 21 HAs as listed in Table S1 were from Toronto Research Chemicals (Downsview, Ontario, Canada). Both UR-EX extraction and UR-CLEAN-II purification kits for QuEChERS extraction were obtained from Yu-Ho Co. (New Taipei City, Taiwan). A Milli-Q water purification system from Millipore Co. (Bedford, MA, USA) supplied deionized water for dilution purposes. For PAHs separation, a DB-5 MS capillary column with dimensions 30 m x 0.25 mm ID and 0.25 μm film thickness was supplied by Agilent Technologies Co (Palo Alto, CA, USA) and an Acquity UPLC BEH C18 column with dimensions 100 x 2.1 mm ID and 1.7 μm particle size was obtained from Waters Co (Milford, MA, USA) for HAs separation. The solvents for HPLC use including acetone, hexane, acetonitrile, and methanol were obtained from Merck Co. (Darmstadt, Germany).

Table S1. Retention time and selected reaction monitoring (SRM) detection parameters of 20 HAs and internal standard (4,7,8-TriMeIQx) by UPLC-MS/MS.

HA compound	Retention time (min.)	Precursor ion (m/z)	Quantitation		Confirmation	
			Product ion (m/z)	Collision Energy (eV)	Product ion (m/z)	Collision Energy (eV)
2-amino-1,6-dimethylimidazo[4,5-b]-pyridine (DMIP)	0.65	163.1	148.1	24	105.09	37
2-aminodipyrido-[1,2-a:3',2'-d]imidazole (Glu-P-2)	1.13	185.1	158.1	25	78.05	37
2-amino-1-methyl-imidazo[4,5-f]-quinoline (iso-IQ)	2.70	199.09	184.12	25	156.10	21
2-amino-3-methyl-imidazo[4,5-f]-quinoline (IQ)	2.71	199.1	184.12	27	157.00	29
2-amino-3-methyl-imidazo[4,5-f]-quinoxaline (IQx)	2.72	200.08	185.12	28	132.14	29
2-amino-3,4-dimethyl-imidazo[4,5-f]- quinoline (MeIQ)	2.49	213.11	198.09	27	145.15	29
2-amino-6-methyldipyrido-[1,2-a:3',2'-d] imidazole (Glu-P-1)	2.68	199.1	92.1	36	172.14	26
2-amino-3,8-dimethyl-imidazo[4,5-f]- quinoxaline (8-MeIQx)	2.65	214.1	131.07	41	173.18	24
2-amino-1-methyl-imidazo[4,5-b]-quinoline (IQ[4,5-b])	2.71	199.11	183.92	27	115.19	46
2-amino-1,6-dimethyl-furo[3,2-e]imidazo[4,5-b]-pyridine (IFP)	2.80	203.08	188.17	25	175.14	22
2-amino-3,7,8-trimethyl-imidazo[4,5-f]- quinoxaline (7,8-DiMeIQx)	2.76	228.1	131.13	40	187.15	25
2-amino-3,4,8-trimethyl-imidazo[4,5-f]- quinoxaline (4,8-DiMeIQx)	2.77	228.1	213.09	26	187.09	23
9H-pyrido[3,4-b]indole (Norharman)	2.82	169.06	115.09	33	89.05	48
2-amino-3,4,7,8-tetramethyl-imidazo[4,5-f]-quinoxaline (4,7,8-TriMeIQx) (IS, internal standard)	2.85	242.13	145.09	42	201.21	26
1-methyl-9H-pyrido[3,4-b]indole (Harman)	2.80	183.09	115.15	34	89.09	49
2-amino-5-phenylpyridine (Phe-P-1)	3.02	171.09	127.13	30	154.07	21
3-amino-1-methyl-5H-pyrido[4,3-b]indole (Trp-P-2)	2.94	198.11	154.14	30	181.08	24
2-amino-1-methyl-6-phenylimidazo[4,5-b]- pyridine (PhIP)	3.05	225.1	210.05	30	140.08	54
3-amino-1,4-dimethyl-5H-pyrido[4,3-b]indole (Trp-P-1)	2.98	212.12	195.14	24	168.09	30
2-amino-9H-pyrido[2,3-b]indole (AαC)	3.11	184.07	140.13	33	167.07	24
2-amino-3-methyl-9H-pyrido[2,3-b]indole (MeAαC)	3.22	198.1	181.14	23	127.13	38

Table S2. Retention time and selected reaction monitoring (SRM) detection parameters of 23 PAHs and internal standard (Triphenylene) by GC-MS/MS.

PAH compound	Retention time (min)	Quantitative ion pair		Qualitative ion pair	
		Precursor ion > Product ion (m/z)	Collision energy (eV)	Precursor ion > Product ion (m/z)	Collision energy (eV)
Naphthalene (NaP)	7.90	128 > 102	20	128 > 78	25
Acenaphthylene (AcPy)	14.5	152 > 151	20	152 > 150	35
Acenaphthene (AcP)	15.6	154 > 153	20	153 > 152	20
Fluorene (Flu)	17.6	166 > 165	20	165 > 164	25
Phenanthrene (Phe)	21.8	178 > 176	35	178 > 152	25
Anthracene (Ant)	22.1	178 > 176	35	178 > 152	25
Fluoranthene (FL)	27.9	202 > 200	40	202 > 201	25
Pyrene (Pyr)	29.7	202 > 200	40	202 > 201	25
Triphenylene (IS, internal standard)	41.1	228 > 226	30	113 > 112	10
Benzo[c]fluorene (BcF)	33.1	216 > 215	20	215 > 213	30
Benzo[a]anthracene (BaA)	41.3	228 > 226	35	113 > 112	15
Chrysene (CHR)	41.6	228 > 226	35	228 > 227	20
5-methylchrysene (MCH)	47.5	242 > 241	40	242 > 239	15
Benzo[b]fluoranthene (BbF)	55.6	252 > 250	40	125 > 124	15
Benzo[j]fluoranthene (BjF)	55.8	252 > 250	40	125 > 124	15
Cyclopenta[c,d]pyrene (CcdP)	58.6	226 > 224	45	113 > 112	15
Benzo[a]pyrene (BaP)	61.2	252 > 250	20	125 > 124	40
Indeno[1,2,3-cd]pyrene (IP)	70.8	276 > 274	45	137 > 136	15
Dibenzo[a,h]anthracene (DBahA)	70.9	278 > 276	40	276 > 274	45
Benzo[ghi]perylene (BghiP)	71.1	276 > 274	45	138 > 137	15
Dibenzo[a,l]pyrene (DBalP)	74.9	302 > 300	40	150 > 149	20
Dibenzo[a,e]pyrene (DBaeP)	75.9	302 > 300	40	150 > 149	20
Dibenzo[a,i]pyrene (DBaiP)	76.4	302 > 300	40	150 > 149	20
Dibenzo[a,h]pyrene (DBahP)	76.8	302 > 300	40	150 > 149	20
